# Implementation of integrated geriatric care at a German hospital: a case study to understand when and why beneficial outcomes can be achieved

**DOI:** 10.1186/s12913-017-2105-7

**Published:** 2017-03-07

**Authors:** Loraine Busetto, Jörn Kiselev, Katrien Ger Luijkx, Elisabeth Steinhagen-Thiessen, Hubertus Johannes Maria Vrijhoef

**Affiliations:** 1 Tranzo Scientific Center for Care and Welfare, Tilburg University, Warandelaan 2, 5037 AB Tilburg, The Netherlands; 2 Geriatrics Research Group, Charité Universitätsmedizin Berlin, Berlin, Germany; 3 Panaxea B.V., Amsterdam, The Netherlands; 4 Department of Patient & Care, Maastricht University Medical Center, Maastricht, The Netherlands; 5 Department of Family Medicine and Chronic Care, Vrije Universiteit Brussel, Brussels, Belgium

**Keywords:** Integrated care, Geriatric care, CMO model, Chronic care model, Implementation model, Evaluation

## Abstract

**Background:**

Many health systems have implemented integrated care as an alternative approach to health care delivery that is more appropriate for patients with complex, long-term needs. The objective of this article was to analyse the implementation of integrated care at a German geriatric hospital and explore whether the use of a “context-mechanisms-outcomes”-based model provides insights into when and why beneficial outcomes can be achieved.

**Methods:**

We conducted 15 semi-structured interviews with health professionals employed at the hospital. The data were qualitatively analysed using a “context-mechanisms-outcomes”-based model. Specifically, mechanisms were defined as the different components of the integrated care intervention and categorised according to Wagner’s Chronic Care Model (CCM). Context was understood as the setting in which the mechanisms are brought into practice and described by the barriers and facilitators encountered in the implementation process. These were categorised according to the six levels of Grol and Wensing’s Implementation Model (IM): innovation, individual professional, patient, social context, organisational context and economic and political context. Outcomes were defined as the effects triggered by mechanisms and context, and categorised according to the six dimensions of quality of care as defined by the World Health Organization, namely effectiveness, efficiency, accessibility, patient-centeredness, equity and safety.

**Results:**

The integrated care intervention consisted of three main components: a specific reimbursement system (“early complex geriatric rehabilitation”), multidisciplinary cooperation, and comprehensive geriatric assessments. The inflexibility of the reimbursement system regarding the obligatory number of treatment sessions contributed to over-, under- and misuse of services. Multidisciplinary cooperation was impeded by a high workload, which contributed to waste in workflows. The comprehensive geriatric assessments were complemented with information provided by family members, which contributed to decreased likelihood of adverse events.

**Conclusions:**

We recommend an increased focus on trying to understand how intervention components interact with context factors and, combined, lead to positive and/or negative outcomes.

**Electronic supplementary material:**

The online version of this article (doi:10.1186/s12913-017-2105-7) contains supplementary material, which is available to authorized users.

## Background

Over the past decade, many health systems have implemented integrated care as an alternative approach to health care delivery that is more appropriate for patients with complex, long-term needs. The World Health Organization (WHO) defines integrated care as “the management and delivery of health services such that people receive a continuum of health promotion, health protection and disease prevention services, as well as diagnosis, treatment, long-term care, rehabilitation, and palliative care services through the different levels and sites of care within the health system and according to their needs” [1]. As such, integrated care interventions often include changes to the health system, use of community resources, patient-provider relationships, care process design, communication infrastructures and the ways in which health professionals deliver care [2–4]. This is expected to lead to improved population health, patient experiences and cost-efficiency [5–8], a trio of goals commonly referred to as the Triple Aim [9].

When persons fall ill at an old age, they are often referred to as geriatric patients, even though exact definitions differ. Generally speaking, geriatric patients suffer from geriatric conditions, that is, a collection of signs and symptoms which are common in older patients [10, 11]. These often include incontinence, falls, malnutrition or low body mass index, dehydration, constipation, depression, pressure ulcers, mobility disability, dizziness, vision impairment, hearing impairment, cognitive impairment, delirium, insomnia, and polypharmacy [10, 12–17]. As these conditions are often not associated with a specific disease and therefore fall outside the scope of traditional disease-focussed models of care delivery, it has been argued that integrated care interventions are especially important for this target population [11–13, 18]. However, so far the evidence on the effectiveness of integrated care for people with geriatric conditions has been mixed. While some interventions were found to have contributed to a reduction in symptoms, emergency department visits, acute hospital admissions and hospital bed days [19, 20], other interventions showed no improvements in length of hospital stay, use of care, prevention of adverse outcomes, health status and costs [20–25]. In addition to this heterogeneity in outcomes, there was also a considerable variation in the interventions themselves, which ranged from telehealth education, discharge planning and community support, and multidisciplinary pathways to integration of acute, chronic and social care.

Rather than assessing whether integrated care for geriatric conditions “works”, it should be explored why and in which cases some interventions do, while others do not. This requires a focus on the implementation of an intervention, including which type of intervention is implemented, how it is affected by the context in which it is implemented, and to which outcomes it contributes [26–28]. In order to gain more insights into the implementation of integrated care for people with geriatric conditions, the first objective of our study is to describe the implementation of an integrated geriatric care intervention at a German geriatric hospital. This case was selected as case study of integrated care implementation within the scope of a comparative European project [29]. To facilitate the analysis of the case study, we make use of a CMO (context-mechanisms-outcomes)-based model, which assumes that interventions have beneficial outcomes when they introduce appropriate mechanisms in the appropriate social and cultural contexts [27, 30]. The second objective of this study is to explore whether the application of this model provides insights into when and why beneficial outcomes can be achieved.

## Methods

### Case selection

Within the scope of a European research project, a German geriatric hospital was identified as a case study of integrated care implementation for patients with geriatric conditions. Within the project, four case studies were conducted on integrated care for two specific chronic conditions (i.e. diabetes and chronic obstructive pulmonary disease) and two groups of conditions (i.e. mental health and geriatric conditions), with the aim of identifying what constitutes good quality integrated care provision [31]. The German case site was one of the first geriatric hospitals in Germany intentionally organised as a multidisciplinary hospital with an integrated care approach. It includes a geriatric hospital with more than 150 beds, a day clinic and a nursing home specialised in dementia care. In this article, we focus specifically on the geriatric hospital. Its patient population consists of patients with complex, multiple age-related conditions that are in temporary need of acute care before they can be discharged or transferred to a long-term care facility [32]. Care for geriatric patients is delivered by a geriatric team led by a geriatric physician, who have weekly team meetings and perform standardised comprehensive geriatric assessments. Patients stay at the hospital for up to 21 days, depending on their health status and potential for rehabilitation. Subsequently, they are discharged to their home setting or transferred to a nursing home for long-term care.

### CMO-based approach

**Fig. 1:**
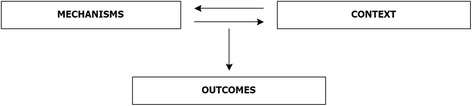
Relationship between mechanisms, context and outcomes

As research informed by the CMO Model is best equipped to answer ‘how’ and ‘why’ questions in combination with ‘what has been achieved’ questions, we use the CMO Model as an umbrella framework for the collection, analysis and interpretation of data [27]. It should be noted that even though the CMO Model suggests the order “context, mechanisms, outcomes”, in the following we will discuss the elements in the order “mechanisms, context, outcomes”. The reason for this is that in our understanding, the mechanisms of an intervention are introduced, then they encounter barriers and facilitators in a specific setting, which combined with the mechanisms, lead to certain outcomes. This relationship is shown in Fig. 1.

*Mechanisms* were defined as the different components of the integrated care intervention and categorised according to Wagner’s Chronic Care Model (CCM). According to the CCM, improvements in integrated care for chronic conditions require changes in six components (health system, self-management support, delivery system design, decision support, clinical information system and community) [4]. Interventions targeting at least two of these components are generally considered integrated care [3, 30, 33–36]. *Context* is understood as the setting in which the mechanisms are brought into practice and described by the barriers and facilitators encountered in the implementation process. We define barriers and facilitators as those factors that either hinder or foster the implementation of integrated care interventions in practice. These were categorised according to the six levels of Grol and Wensing’s Implementation Model (IM): innovation, individual professional, patient, social context, organisational context and economic and political context [37]. During the analysis, the level “health system context” was added and the level “economic and political context” was changed to “economic, political and legal context” to make it more explicit that the legal/regulatory dimension was also covered. *Outcomes* are defined as the effects triggered by mechanisms and context. These were categorised according to the six dimensions of quality of care as defined by the WHO, namely effectiveness, efficiency, accessibility, patient-centeredness, equity and safety [38]. During the analysis, we added satisfaction as additional dimension to accommodate the interview data on care giver and patient satisfaction. The development and final version of the model are described elsewhere [39].

### Data collection and analysis

After receiving approval by the ethical review committee of Charité Universitätsmedizin Berlin, potential interviewees were contacted personally (face-to-face) by one of the researchers (JK). In order to achieve data saturation, ensure diversity in professional backgrounds and include different perspectives on the integrated care intervention, we aimed for a sample size that included at least two interviewees from each of the seven professional group represented in the multidisciplinary team (i.e. medical doctors, occupational therapists, neuropsychologists, physical therapists, nurses, speech therapists and social workers). After conducting interviews with at least two respondents from each professional group, the researchers concluded that it was indeed likely that no new information would be collected by adding new respondents from the same groups, and data saturation was thus achieved. However, it was deemed likely that adding respondents from other professional groups would have led to new information, but this was not feasible for practical reasons (i.e. high workload). The final response rate was 75%. Fifteen interviews of approximately 1 h were conducted with four medical doctors, four occupational therapists, three neuropsychologists, two physical therapists and two nurses. Speech therapists and social workers were not represented in the sample. During the interviews, a topic list was used that focussed on the various components of the integrated care intervention, the barriers and facilitators to its implementation, and the outcomes achieved because of the intervention (Additional file 1). This topic list had been developed by a consortium of researchers involved in the European comparative project and tested and used in a similar case study on integrated diabetes care in the Netherlands [30]. The interviews were conducted face-to-face either at the hospital or the researcher’s office. Only the interviewer (JK) and the interviewee were present during the interviews. JK is university educated to a Master’s degree and has extensive previous experience in conducting semi-structured interviews. All interviews were audio-taped and transcribed verbatim afterwards. Due to the strict anonymity requirements by the ethical review committee, there were no records of the interviewees’ identities, and it was therefore not possible to perform member checks as a quality assurance measures. This also meant that no demographic data (such as respondents’ age or gender) were collected.

Data analysis was performed by two researchers (JK, LB). JK had conducted the interviewes and knew the interviewees and their work environment well. LB was an outsider who had not met the interviewees and was not familiar with their work environment. The initial coding framework was an adapted version of the coding framework from the Dutch case study on integrated diabetes care and included codes related to the different CCM components, the role of the interviewee, the patient population, the implementation of the intervention (including the foundation of the hospital, changes over time, barriers and facilitators to the execution of the intervention), transfers to and from the geriatric hospital, and middle and senior management and leadership. Paper-based coding of the interview transcripts was performed independently by two researchers (LB, JK). After five interviews, the sections for which a certain code was identified, were summarised and translated from German to English. The translated summaries were transferred to a tabular form of the coding framework (i.e. the coding table). The coding table was compared to and adapted according to the second researcher’s (JK) paper based coding. The adapted coding table was complemented by the next five interviews’ paper based codes. This was repeated a third time until all paper based coding was summarised in the coding table. Throughout this process, the framework of available codes was adapted when necessary after discussion among the two coders (LB, JK). Based on the information summarised in the coding table, the main mechanisms, context factors and outcomes were identified, described and visualised according to the CMO-based model described above. This resulted in an overview of the mechanisms, context factors and outcomes, as well as three clusters of their interrelationships described around the three main intervention components.

## Results

We found the integrated care intervention to consist of three main components, namely a specific reimbursement system (called “early complex geriatric rehabilitation”), multidisciplinary cooperation and comprehensive geriatric assessments (see Table 1). The implementation of these components was hindered by barriers such as a sub-optimal documentation system; patients with increasingly complex conditions; high workload, lack of inter-organisation infrastructure; and administrative obligations. The implementation was facilitated by family member involvement; informal cooperation structures; and also administrative obligations (Table 2). In combination, mechanisms and context factors contributed to negative outcomes such as less care provided to the patients; overuse, underuse and misuse of health services; unnecessary incurrence of costs; waste in workflows (i.e. non-value-adding activities [40, 41]); less focus on the patient instead of administrative obligations; less family involvement; increased likelihood of adverse events or medical mistakes; revolving door effect and frustration among staff. Positive outcomes included better understanding of colleagues’ expertise; continuity in care provision; more care provided to the patient; financially advantageous reimbursements; faster information exchange; more focus on the patient instead of administrative obligations; holistic view of the patient; improved transparency; decreased likelihood of adverse events or medical mistakes; appreciation by staff; and appreciation by patients (Table 3).

**Table 1:** Overview of the mechanisms of the integrated care intervention

CCM component	Mechanisms
Health system	Early complex geriatric rehabilitation
Self-management support	n/a
Delivery system design	Multidisciplinary cooperation
Decision support	Comprehensive geriatric assessment
Clinical information system	n/a
Community	n/a

**Table 2:** Overview of the context of the integrated care intervention

IM Level	Barriers	Facilitators
Innovation	Documentation system	n/a
Individual professional	n/a	n/a
Patient	Increasingly complex conditions	Family member involvement
Social context	n/a	n/a
Organisational context	High workload	Informal cooperation structures
Heath system context	Lack of inter-organisational infrastructure	n/a
Economic, legal and political context	Administrative obligations	Administrative obligations

**Table 3:** Overview of the outcomes of the integrated care intervention

WHO Dimension	Negative outcomes	Positive outcomes
Effectiveness	•Less care provided to patients	•More care provided to patients •Better understanding of colleagues’ expertise •Continuity in care provision
Efficiency	•Overuse, underuse, misuse •Waste in workflows	•Financially advantageous reimbursements •Faster information exchange
Accessibility	n/a	n/a
Patient-centeredness	•Less family member involvement •Less focus on patient instead of administrative considerations	•More focus on patient instead of administrative considerations •Holistic view of the patient
Equity	n/a	n/a
Safety	•Revolving door effect •Increased likelihood of adverse events or medical mistakes	•Decreased likelihood of adverse events or medical mistakes •Improved transparency
Satisfaction	•Frustration among staff	•Appreciation by staff •Appreciation by patients

In the following section, we describe the interplay of these factors by presenting clusters of mechanisms, context factors and outcomes. For increased clarity, we ordered the clusters around the intervention components, i.e. Cluster 1 around the reimbursement system, Cluster 2 around multidisciplinary cooperation and Cluster 3 around comprehensive geriatric assessments.

### Cluster 1: Early complex geriatric rehabilitation

The care patients receive at the geriatric hospital is reimbursed as “early complex geriatric rehabilitation” (in German: geriatrische frührehabilitative Komplexbehandlung (GFK)) *(mechanism)*. This is a technical term that describes a specific reimbursement option within the German system of disease related groups (G-DRG) [42–45]. The G-DRGs are an obligatory classification system used by hospitals to receive bundled reimbursements for the treatment of similar groups of patients. Patients are classified into specific DRGs based on demographic data, primary and secondary diagnoses and medical procedures. The procedure applicable to geriatric care is the GFK which is meant for geriatric patients in temporary need of acute care (OPS 8-550). In order to be eligible for reimbursement under the GFK framework, the geriatric hospital must fulfil certain criteria. Among other criteria, they must show that the patient is a geriatric patient (i.e. multimorbid, often 70 years and older), care must be provided by a geriatric team led by a geriatric physician, and standardised comprehensive geriatric assessments as well as weekly team meetings must take place. Additionally, patients staying at the geriatric hospital for a certain number of days must receive a certain number of therapy sessions. For example, patients staying 14 days need to receive 20 therapy sessions, and patients staying 21 days require 30 therapy sessions. If the patient does not need complex care, they can be discharged earlier without the number of required therapy units. In this case, the GFK framework does not apply and the patient is classified according to his or her primary conditions within the G-DRG system. However, for the geriatric hospital, reimbursements as GFK are financially advantageous compared to the regular rates *(outcome)*.

One of the characteristics of the GFK is its inflexibility regarding the number of treatment sessions that have to be provided to each patient. Given the vast differences in rehabilitation potential of the patients, this standardisation leads to overuse of services by some patients, and under- and misuse of services by others *(outcome)*. This leads to frustration when the health professionals feel that treatments are provided to patients who cannot benefit much from these treatments, at the expense of other patients who could benefit greatly but who have reached their maximum number of treatments *(outcome)*. As mentioned above, the GFK framework offers advantageous reimbursement rates when patients stay at the geriatric hospitals for a minimum of 14 days and receive 20 therapy sessions, or 21 days and 30 therapy sessions. However, day 14 and 21 are cut-off points, and there is no financial incentive for hospitals to keep patients for more than 14 days (but less than 21), or more than 21 days when the required number of therapy sessions has been provided. Instead, when patients stay longer than these cut-off days, hospitals incur the costs of having patients at the hospital without receiving an additional reimbursement. As one interviewee observed:

*“I would say the moment when everything changed was when the disease-related groups were introduced, and brought with it the commercialisation of the health care sector.”*

Interviewee 1

As a consequence, interviewees noticed a change in the care planning approach. One example given in this context was that even if all health professionals agreed that a few more therapy units would greatly improve the patient’s health status, the patient would still have to be discharged at the end of the 21 day period. In this sense, the hospital’s financial considerations were more decisive for the patients’ care trajectories than the patients’ needs and wishes, and as a consequence, patients received less or even insufficient care *(outcome)*. The latter contributed to a revolving door effect, meaning that patients were re-admitted soon after discharge. This transfer to and from different settings was potentially dangerous to patient health, especially for frail older people *(outcome)*. The negative effects of the increased focus on the length of stay were further exacerbated by the fact that the patient population in the geriatric hospital was characterised by more and increasingly complex conditions *(context).* This made it less likely that an appropriate amount of care could be realised within the same timeframe *(outcome).*

Another characteristic of the GFK is the fact that it is not a full-range treatment on its own, but an intermediate stop between a previous location (home or care facility) and the next location (also home or care facility). However, there is a lack of a general infrastructure (including IT infrastructure) to support inter-organisational cooperation between the geriatric hospital and referring/admitting institutions and the ambulatory sector *(context)*. For example, sometimes patients arrive at the geriatric hospital without the necessary information on their medical condition:

*“Patients arrive without a letter of referral. That’s an absolute no-go, it must not happen, even if it’s only a short letter with the most essential diagnoses. But it should never happen that a multimorbid patient arrives here without a referral letter, with only a short consultation between the nurses. Well, what can you do? You pick up the phone and call a number only to hear that your colleague is currently in surgery and there’s no one available to give at least a short summary of the patient. That’s bad style, but it happens on a daily basis. The time we spend on the phone, to get to know at least something, it’s terrible.”*

Interviewee 2

**Fig. 2:**
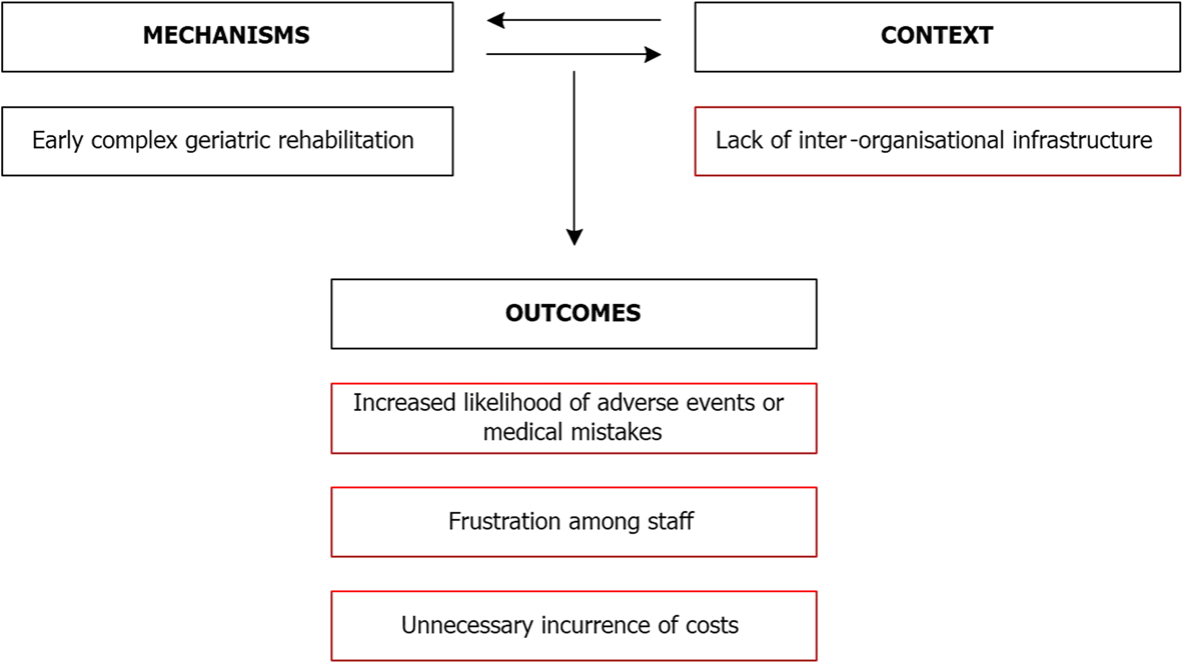
Example of the interplay between mechanism, context and outcomes in Cluster 1. *Red boxes* indicate barriers and negative outcomes

This lack of information is time-consuming and frustrating for health professionals *(outcome)*. Moreover, it is potentially dangerous for the patients, since health professionals have to rely on incomplete information to make medical decisions *(outcome)*. Another example of the problematic inter-organisational infrastructure is the fact that patients should not be discharged from the geriatric hospital on a Friday because it is often not clear how they will get their medicines until Monday or how they are otherwise supported at their homes. Often this entails a longer length of stay at the geriatric hospital over the weekend and thereby increased costs for the hospital, without adding any benefit for the patient who does not receive additional treatment sessions in the weekends *(outcome)*. As an example of the interplay between mechanism, context and outcomes in Cluster 1, the influence of the lack of inter-organisational infrastructure is portrayed in Fig. 2.

### Cluster 2: Multidisciplinary cooperation

The geriatric hospital has multidisciplinary staff consisting of doctors, nurses, occupational therapists, physical therapists, speech therapists, neuropsychologists and social workers. Depending on the patient’s needs, different combinations of health professionals work together. For example, physical therapists perform certain measures that make it possible for occupational therapists to mobilise that part of the patient’s body. For stroke patients with cognitive impairments, it makes sense for the occupational therapists to cooperate primarily with the neuropsychologists. As one interviewee put it:

*“Basically it’s a whole network of staff from different professional groups who are linked to one another and who communicate so that the patient is cared for in an optimal way.”*

Interviewee 3

The multidisciplinary team meets regularly during daily morning meetings and weekly team meetings. During the daily morning meetings, new patients are introduced by the doctor and nurses from the night shift who describe whether there were any events during the past shifts. The weekly team meetings are obligatory under the GFK framework and are led by the ward doctor. Here, the patients’ advances during the past week are discussed from the professional groups’ different perspectives. At the end of the discussion a therapy plan for the patient for the next week is agreed on and the possibilities for discharge or transfer to a nursing home are discussed. One interviewee framed it the following way:

*“They [the team meetings] are very important and they are what distinguishes us from acute medicine.”*

Interviewee 4

Interviewees have indicated that they enjoyed the multidisciplinary cooperation, mainly because the flat hierarchical structures led to a lot of independence and accountability of each member of the team *(outcome)*. Formal meetings were seen as an important factor in improving the efficiency of the information exchange and thereby saving time for the health professionals *(outcome)*. Moreover, interviewees indicated that because of the team meetings, health professionals already had a lot of information about the patients and therefore did not need to ask the same questions several times, which otherwise would have led to frustration among the patients and providers *(outcome)*. An additional benefit was seen in the deeper understanding of the other health professionals’ perspectives and work with the patient, including the interpretation of assessments *(outcome)*.

However, interviewees indicated that the current documentation system acted as a barrier to their cooperation *(context).* They indicated that the documentation system was too old, too slow, and too unreliable, because it broke down frequently. Moreover, not all health professionals could see all data produced by the other professions, and not all relevant information could be documented with the system. Overall, these problems resulted in considerable frustration among staff and waste in workflows *(outcome)*.

*“I have to log on and put in the (patients’) diagnoses and that’s so time-consuming. You can really watch the minutes go by before you can start to enter the code. And we’re really paid too well for this, you know. It’s just a total waste of time.”*

Interviewee 5

Over time, many informal cooperation structures were established at the geriatric hospital as a workaround to the limited number of weekly team meetings and impediments due to the lack of a proper IT system *(context)*. For example, direct communication in passing between health professionals effectively replaced the gathering of information via the patient record. Furthermore, it made it possible to re-adjust strategies in between the weekly team meetings. As such, informal cooperation led to faster information exchange *(outcome)*. Moreover, interviewees indicated that team meetings often centred on organisational issues for fulfilling prerequisites of the GFK framework. The informal cooperation allowed the health professionals to discuss the patient instead of administrative obligations *(outcome)*.

**Fig. 3:**
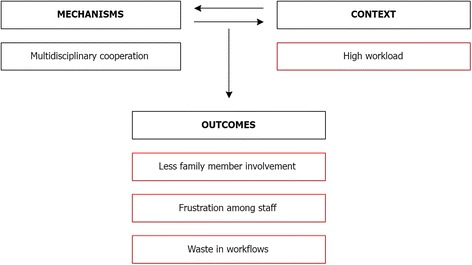
Example of the interplay between mechanism, context and outcomes in Cluster 2. *Red boxes* indicate barriers and negative outcomes

Interviewees indicated that they experienced a high workload at the geriatric hospital *(context)*. This meant that they did not have enough time to talk to their colleagues, which led to the fact that the treatment approaches of the different health professionals were not always well coordinated and aligned to one another, which contributed to waste in workflows *(outcome)*. Moreover, staff felt that they did not spend enough time with the patient either, which led to frustration *(outcome)*. High workload was also seen as a major barrier to increased family involvement and information provision *(outcome)*. As an example of the interplay between mechanism, context and outcomes in Cluster 2, the influence of a high workload on multidisciplinary cooperation shown in Fig. 3.

Moreover, interviewees reported that administrative obligations multiplied over the past 10–15 years *(context)*. This contributed to a shift of focus during the team meetings, away from discussing the patients as a multidisciplinary team, and towards making sure that patients received the obligatory number of sessions or that length of stay would not be exceeded *(outcome)*. Interviewees realised that documentation was important in terms of transparency *(outcome)*. However, as long as this increasing imbalance towards documentation was not compensated by more staff to spend time with the patient, less care would be provided to the patients *(outcome)*. This also led to frustration by staff who felt that their time is not spent on what should be their most important task, namely taking care of their patients *(outcome)*. Finally, interviewees reported that when family members were included in the care process and educated on how to take care of the patient *(context)*, this helped to decrease the workload of the nurses, which meant that they could better cooperate with their colleagues and deliver more care to the patient *(outcome)*. Family member involvement also enabled them to continue the care once the patient was discharged *(outcome)*.

### Cluster 3: Comprehensive geriatric assessments

At the geriatric hospital, each patient is assessed by all health professionals during in-take and before discharge *(mechanism)*. Certain parts of these assessments are obligatory, while others depend on the mobility or cognitive abilities of the patient. All assessments are documented in the documentation system, which is a necessary condition for receiving reimbursements via the GFK framework. They are also used as a basis for the discussions in the team meetings and further care planning. Interviewees indicated that the comprehensive geriatric assessments made it possible to include various perspectives and thereby different interpretations of the patient’s situation.

*“I think that’s partly the advantage of using multiple pairs of eyes to assess the patient, that it allows for different perspectives and therefore different interpretations. It’s often only by taking into account different types of information that you find the right way to the patient.”*

Interviewee 6

**Fig. 4:**
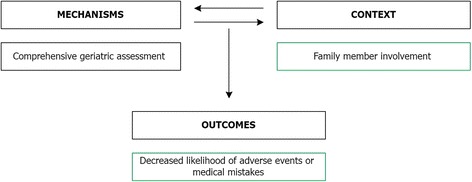
Example of the interplay between mechanism, context and outcomes in Cluster 3. *Green boxes* indicate facilitators and positive outcomes

In doing so, they were seen as an enabler of a holistic view of the patient as a whole person instead of separate parts of the body or illnesses that must be treated *(outcome)*. Moreover, interviewees pointed out that this type of cooperation between all health professionals compensated for the loss of the patient as an information carrier (e.g. due to cognitive or speech impairments) and thereby helped to prevent adverse events or medical mistakes *(outcome)*. The involvement of family members made it possible to complement the comprehensive geriatric assessments with other sources of information *(context)*. This was necessary because these assessments can never capture all relevant information, and they are sometimes invalidated by the patients themselves who are not entirely honest or try to embellish their situation. Especially in the case of chronically ill patients, family members hold valuable information which often helps to prevent mistakes or adverse events from (re-)occurring *(outcome)*. As an example of the interplay between mechanisms, context and outcomes in Cluster 3, the influence of family member involvement on the comprehensive geriatric assessments is presented in Fig. 4.

#### Missing CCM components

Based on the interviews, we did not find self-management support, a clinical information system and use of community resources to be part of the intervention. The interviewees rarely mentioned self-management support as a topic, even though some health professionals described a patient-centred approach to care planning for their own therapy sessions. For example, one occupational therapist described how he discusses goals and priorities with the patients to determine the care plan:

*“I look at: what can the patient do? What can’t he do yet? And then I discuss with him: what are his goals? What does he want to achieve with me and with the therapy? Then I give the patient some time and say: think about it before the next session.”*

Interviewee 7

However, no structured programs or approaches to self-management support or the involvement and training of family members existed. The IT system used in the geriatric hospital was generally seen as a digital documentation system or administrative tool rather than a real-time information system from which information was actively accessed. Some health professions stated that the system was mainly used to fulfil the documentation requirements for the reimbursement within the GFK framework. With regard to the use of community resources, most interviewees were aware that in the discharge of the patient from the geriatric hospital to the patient’s home setting, there are different actors who play a role and who can be involved to optimise the patient’s situation. However, we found no evidence of how community resources were mobilised or linked with the hospital to this purpose.

## Discussion

This study presented an analysis of the implementation of integrated geriatric care at a German geriatric hospital by identifying the main intervention components, how they were affected by the context in which they were implemented, and to which outcomes they contributed. We made use of a CMO-based model to create clusters describing the interplay of mechanisms, context factors and outcomes.

We found the integrated care intervention at the geriatric hospital to consist of three main components, namely a specific reimbursement system, multidisciplinary cooperation and comprehensive geriatric assessments. Reimbursements as GFK *(health system)* are financially advantageous for the geriatric hospital *(efficiency).* Moreover, the inflexibility of the GFK framework regarding the obligatory number of treatment sessions as well as its focus on the length of stay contributed to less care delivered to the patient *(effectiveness*), overuse, underuse and misuse of health services *(efficiency)*, less focus on the patient instead of administrative obligations *(patient-centeredness)*, a revolving door effect *(safety),* and frustration among staff *(satisfaction).* This was further exacerbated by a patient population with increasingly complex conditions *(patient).* The execution of the GFK framework in practice was impeded by the lack of inter-organisational infrastructure *(health system context),* which contributed to unnecessary incurrence of costs *(efficiency),* an increased likelihood of adverse events or medical mistakes *(safety),* and frustration among staff *(satisfaction).* These findings resonate with Kolb et al.’s evaluation of the GFK 10 years after its inception, from the perspectives of the German Federal Association of Geriatrics (BVG), the German Health Insurance Medical Service (MDK) and the National Association of Statutory Health Insurance Funds (GKV Spitzenverband) [32]. The authors also found evidence of a lacking inter-organisational structure, which was not in line with the overarching goals of comprehensive geriatric care, namely a transsectoral, interlinked, and therefore holistic approach to geriatric care. Moreover, they found the financial disincentives inherent to the GFK framework to have led to an increased focus by hospitals and financiers on the cut-off points for eligibility. On the one hand, they saw financial incentives for hospitals to keep patients for longer than 14 days to be eligible for the financially advantageous framework. But at the same time there were financial incentives for the financiers to dispute the necessity for ambulatory care for longer than 14 days so as to make hospitals ineligible for the framework, which is financially disadvantageous to the financiers. Kolb et al. also found that hospitals tended to limit their services to the minimum requirements stipulated in the framework. The authors criticised that there is currently no quality system to counter-balance this trend. Additionally, they raised doubts about whether the decision which and how much care is delivered to the geriatric patients was determined by their need or rather the wish by the hospital to optimise the reimbursements. This is in line with our finding that certain aspects of the GFK framework contribute to the over- and under-provision of health services to the patients and increased focus on administrative obligations at the expense of patient-centeredness.

Multidisciplinary cooperation *(delivery system design)* contributed to a better understanding of other health professionals’ expertise *(effectiveness),* faster information exchange *(efficiency)*, and appreciation by staff and patients *(satisfaction).* On the one hand, the execution of multidisciplinary cooperation in practice was impeded by the documentation system *(innovation),* high workload *(organisational context),* and administrative obligations *(economic, legal and political context).* These barriers contributed to less care provided to the patients *(effectiveness),* waste in workflows *(efficiency),* less focus on the patient instead of administrative considerations, less family member involvement *(patient centeredness)*, and frustration among staff *(satisfaction).* On the other hand, multidisciplinary cooperation was facilitated by family member involvement *(patient)*, informal cooperation structures *(organisational context)*, and administrative obligations *(economic, legal and political context).* These positive context factors contributed to a better understanding of one’s colleagues’ expertise, continuity in care, more care provided to the patients *(effectiveness),* faster information exchange *(efficiency),* more focus on the patient instead of administrative considerations *(patient-centeredness)*, improved transparency *(safety),* and appreciation by staff and patients *(satisfaction).* It is difficult to compare these findings to other studies on multidisciplinary cooperation within integrated geriatric care interventions, as these are sparse, especially comprehensive qualitative ones. A review of multidisciplinary collaboration within the scope of collaborative care management models found mixed results for mortality, clinical, functional and social outcomes, utilisation of medical services, quality of life, activities in daily living and satisfaction with care. However, the authors concluded that relationships between teamwork and patient outcomes were difficult to assess with randomised controlled trials (RCT) [46]. A qualitative study on interdisciplinary team collaboration during discharge of depressed older people identified the lack of effective team leadership, the need to change the delivery system, and enhancing self-management support including family member involvement as important context factors. However, the impact of these context factors on specific outcomes was not explicitly studied or discussed [47]. A qualitative study on integrated end of life care for people with advanced dementia did explicitly focus on the context, mechanisms and outcomes of the intervention, but used a different operationalisation of the concepts. Their study underscored the importance of multidisciplinary cooperation in integrated care and the danger of weighing financial efficiency over person-centeredness [48].

Comprehensive geriatric assessments *(decision support)* contributed to a holistic instead of disease-focused view of the patient *(patient-centeredness)* and a decreased likelihood of adverse events or medical mistakes *(safety)*. The achievement of the latter outcome was further enhanced by family member involvement *(patient).* As in the case of multidisciplinary cooperation, (qualitative) research on comprehensive geriatric assessments within the scope of integrated care interventions is still relatively sparse. A recent scoping review of interdisciplinary geriatric consultation teams in acute care hospitals found that the structure and processes of care provided by these teams were highly heterogeneous [49]. However, the relationship of these different intervention types to context factors or outcomes was not studied or discussed. A qualitative study on the facilitating and impeding factors to the implementation of geriatric assessment and decision support in residential care homes found positive opinions of staff and management, continuing support of staff and the availability of sufficient computer equipment to be necessary conditions for intervention adoption [50]. However, the study did not link the intervention itself and the context factors affecting its implementation to outcomes achieved, which again makes it difficult to compare findings. A systematic review of in-patient comprehensive geriatric assessments found positive outcomes, including an increased chance of patients living at home in the long term, especially for ward-based management units [51]. Another review of effectiveness of gerontologically informed nursing assessment and referral interventions for older people in the emergency department reported mixed results for patient and health systems outcomes. Here, too, the authors stressed that testing of complex interventions in RCTs was inherently problematic [52].

We concluded that evidence of the self-management support component was largely absent at the geriatric hospital. A mixed methods study among older people with long-term conditions found self-management support to be associated with continued active participation and completion of a strength and balance intervention [53]. However, in their study among chronically ill older adults with complex medical needs, Gerber et al. cautioned that effective self-management support should be attuned to the older people’s ability to self-manage, which may be hindered by factors such as depression, health literacy, or hearing impairments [54]. We found no clinical information system at the geriatric hospital. In their 2003 study on medical informatics in geriatrics, Nebeker, Hurdle and Bair predicted that barriers to information exchange would decrease while the quality and relevance of exchanged information would increase [55]. Ten years after this prediction we would have to conclude that this may have been too optimistic, at least for the German healthcare sector in which barriers to (electronic) information exchange between organisations, providers and/or patients continue to exist [56–59]. Unfortunately, this is not a German problem, as a systematic review of the barriers to the acceptance of electronic medical records by physicians showed. Based on studies conducted in the United States, Canada, Israel, Norway, and Ireland, the authors found financial, technical, time-related, psychological, social, legal, organisational and change process-related barriers that contributed to low adoption rates of IT systems [60]. At the time of writing, however, a new digital information system has been implemented at the geriatric hospital which would provide an interesting case for further investigation in light of these barriers as well as the findings of the current study. Finally, we did not find evidence of the use of community resources, but this could be due to the fact that no social workers were interviewed, i.e. the group responsible for discharge arrangements. In general, the use of community resources might be impeded by the difficult regulatory framework for cross-sectoral health care in Germany [59, 61–63]. Again, this is not an exclusively German problem, as other health systems in- and outside Europe also experience considerable barriers to inter-organisational or cross-sectoral cooperation. For example, the European Union financed the HANDOVER project, conducted in Italy, the Netherlands, Poland, UK, Spain, and Sweden, to improve transitions at the primary care-inpatient interface [64]. In their study into hospital discharge of older patients to primary health care in the Norwegian context, Dahl et al. found communication barriers across care levels, despite the use of intermediate care hospital specifically to smooth the transition from secondary to primary care [65]. These examples show that improvement efforts are still ongoing and that more insights are needed in the reasons why these systemic obstacles persist.

### Appraisal of the CMO-based model

In this study, we explored the usefulness of our CMO-based approach for studying when and why an intervention “works”. Our analysis has shown how certain components of the intervention itself have contributed to negative or positive outcomes, and how in other cases, the execution of specific components of the intervention was facilitated or hindered by context factors exterior to the intervention. We believe that this approach indeed allows for more targeted improvements than only investigating whether certain outcomes have improved or not. First, because it allows for the finding of positive as well as negative results, even for the same category, rather than an aggregate estimate or net effect of the intervention. For example, we found more and less focus on patients instead of administrative obligations as outcomes for patient-centeredness, and more and less care provided to patients as outcomes for effectiveness. It is more useful to know both the negative and positive sides of these outcomes, rather than knowing that overall, there is neither a significant positive or negative effect of the intervention. Second, our approach makes the reasons for the achievement of positive and negative outcomes visible. For example, it was shown that due to the high workload, the members of the multidisciplinary team did not have time to truly cooperate with each other, which prevented positive outcomes and contributed to negative ones. Knowing these intricacies is more useful for designing plans for improvement than having to conclude that the multidisciplinary approach did not lead to improved outcomes. A third advantage of our CMO-based approach is that the model helps to not only come up with a list of isolated barriers and facilitators, but instead, to consider their interplay with the mechanism and outcomes, e.g. with which aspects of the integrated care intervention do they interact, which do they impede or enhance, and to which outcomes does this lead. In doing so, our CMO-based approach has proven to be a valuable instrument for answering questions of when, why and how an intervention can contribute to positive outcomes.

### Limitations

Our study is subject to several limitations which should be taken into consideration. First, our findings are based on a convenience sample which did not include patients or their family members, speech therapists and social workers, as well as other stakeholder groups from outside the multidisciplinary team, such as IT experts, hospital administrators or financiers. Including patients in our interviews would have given us the opportunity to evaluate the intervention directly from patients’ perspectives rather than relying on what the health professionals thought the patients’ evaluation would be. Moreover, our findings show the involvement of family members as a facilitator for multidisciplinary cooperation as well as the lack of family member involvement as a negative consequence of multidisciplinary cooperation when it is not well-executed. The family members’ perspective on how and to what extent they are (or wish to be) involved would provide valuable additional insights to our findings. The inclusion of social workers in our sample could have provided further insights into the use of community resources, or confirmed our impression that this component was largely absent from the integrated care intervention. Additionally, the inclusion of other professional stakeholders from outside the multidisciplinary team would have enabled us to analyse outside perspectives on salient topics such as the financing of the hospital, regulatory frameworks and personnel distribution. Second, the CMO-based model used in the collection, analysis and interpretation of data assumes that complex, intricate social phenomena and processes can be neatly categorised as either mechanisms, context factors our outcomes. Additionally, the model assumes a linearity and chronology of events that represents a simplification of reality which is not accurate. Instead, a certain factor might be an intervention component which acts as a barrier to the execution of other intervention components, and certain positive outcomes can act as facilitators to other intervention components. However, simplification can be a necessary and useful step when trying to understand the complexities of the real world and thereby making them manageable. Third, being based on the analysis of one case study, our findings are context-specific and cannot be transferred as is to other health systems, cultural backgrounds, care sectors or chronic conditions. However, some of the intervention components and context factors might be similar in other cases and given the detailed explanations of the setting provided here, we believe our results to hold much learning potential for other organisations currently implementing or planning to implement integrated care interventions for geriatric and other chronic conditions. This is especially relevant given the fact that many studies on integrated geriatric care stem from the Dutch context and insights from other settings are still relatively sparse [21–23].

## Conclusions

The current study has identified the main components of an integrated geriatric care intervention at a German geriatric hospital. Moreover, it has traced the relationships between these components, various context factors and the positive and negative outcomes that were achieved. With regard to this specific case, we recommend that policy-makers reconsider the financial disincentives of the funding system, with specific emphasis on how these can be curtailed or at least counter-balanced with appropriate quality assurance measures. In the meantime, we would recommend that managers and practitioners explore context factors at the organisational, social context or individual professional level that could help to keep the negative consequences found in this study in check. Given the fact that not all CCM components were implemented at the geriatric hospital, it is likely that the integrated care intervention has not reached its full potential yet, and we therefore recommend increased efforts to implement structured self-management support strategies and make use of community resources. The impact of the newly implemented clinical information system should be closely monitored to assess whether it has provided a solution to the IT system related barriers reported in this study.

Our study has highlighted several open question to which stakeholders involved in integrated geriatric care urgently need to find answers. For example, it is not clear how the continued need for rehabilitation services can be determined based on other criteria than an inflexible administrative timeframe. We would recommend that the decision of whether or when to discharge a patient should depend primarily on the patients’ goals and rehabilitation potential. The geriatric hospital, for example, already measures, tracks, and discusses these regularly and over time in the patient file and during the team meetings. It is also possible to add a specific measure of patient satisfaction, or other measures to account for the experiences of patients with the services provided, to be measured at discharge. The average patient satisfaction score over time should then not fall below a certain threshold to ensure the quality of services and make sure that patient needs are not ignored at the expense of financial considerations. Putting increased emphasis on these aspects would not be a question of additional resources or activities, but rather of prioritisation, which requires committed leadership. Another question to be addressed concerns how to determine the caseload limit to maximise team functioning and appropriate involvement of team members for individual patient care needs. While answering this question is still work in progress in the practice as well as scientific community [66], a very urgent recommendation would be to listen better to the employees. If all employees indicate that they cannot do their work properly due to the workload, then maybe it is time to make some changes. Additionally, it should be considered how employees can be involved in care planning so that they can assume more autonomy and ownership regarding which and how many tasks to assume. In general, we recommend that academics as well as practitioners, managers and policy-makers involved in the evaluation of complex interventions such as integrated care broaden their focus from merely trying to determine whether an intervention works or not. Given the universally known heterogeneity in outcomes, we should try to understand how the different components of an intervention interact with context factors and, combined, lead to positive and/or negative outcomes. This in depth understanding of the complex and intricate interplay between mechanisms, context and outcomes is a necessary precondition for targeted improvements that can result in real benefits for real people.

## Additional file

Additional file 1: Qualitative interview data. Interview guide for hospital clinicians, nurses, hospital managers, general practitioners, neuropsychologists and therapeutic health professions (physical therapists, occupational therapists and speech therapists). The interview guide (German version) was used for the qualitative data collection. The interviewer used the questions to steer the conversation but was free to diverge from the guide where appropriate. (DOCX 88 kb)

## Abbreviations

CCM: Chronic care model
CMO Model: Context-mechanisms-outcomes model
GFK: Early complex geriatric rehabilitation; in German: geriatrische frührehabilitative Komplexbehandlung
IM: Implementation model
WHO: World Health Organization
