# Supplementary material for: Implementation of integrated geriatric care at a German hospital: a case study to understand when and why beneficial outcomes can be achieved
Source: BMC Health Serv Res. 2017 Mar 7;17:180. doi: 10.1186/s12913-017-2105-7 (PMC5341181; doi:10.1186/s12913-017-2105-7)
Supplement: Additional file 1: — Qualitative interview data. Interview guide for hospital clinicians, nurses, hospital managers, general practitioners, neuropsychologists and therapeutic health professions (physical therapists, occupational therapists and speech therapists). The interview guide (German version) was used for the qualitative data collection. The interviewer used the questions to steer the conversation but was free to diverge from the guide where appropriate. (DOCX 88 kb) [file 12913_2017_2105_MOESM1_ESM.docx]

**Interview guide for**

**hospital clinicians, nurses, hospital managers, general practitioners, neuropsychologists and therapeutic health professions (physical therapists, occupational therapists and speech therapists)**

| **CASE SITE:** |  |
| --- | --- |
| **INTERVIEWEE DETAILS**  **(Include Role/ Responsibilities):** |  |
| **INTERVIEWER:** |  |
| **DATE & LOCATION:** |  |

**Introduction**

Thank you for agreeing to be interviewed as part of Project Integrate, funded by the European Commission. Project Integrate aims at gaining valuable insights into integrated care as a format of service delivery that can improve the response to the challenges faced in Europe’s health and social security systems: an ageing population and an increase in chronic conditions. The evidence gained in this project will contribute to the optimisation of care delivery processes and organisation in different European settings.

[*Please refer to project information sheet for further details*].

This interview is part of the work to seek to examine the practical barriers, facilities to the effective implementation of IC and should take no longer than one hour. In order to ensure your anonymity, you will not sign a consent form. Instead you will implicitly declare your consent by participating in this interview. Please make sure not to mention your own or other people’s names during the interview. Please feel free to ask any additional questions you may have.

| **Definitions** | |
| --- | --- |
| **IC** | Integrated Care |
| **MDT** | Multi-Disciplinary Team(s) |
| **ICT** | Information and Communication Technology |
| **1-5 Scale Measure** | *Measurement scale of 1-5, where 1 can represent: very poor/ or little and 5 can represent: very good/or a lot.* |

**Role of Interviewee**

- Please tell me about your roles and responsibilities within your organization’s IC services?

**Understanding of Integrated Care**

- IC can mean different things to different people. What does it mean to you?
- From your perspective, what do you understand to be the overall aims and objectives of your organisation’s IC work?
- Please state what you believe are the expected benefits of your IC work, in terms of the patients/service users, to your organisations, and to the overall healthcare system?

**The Client/Patient Group**

- How would you summarise the type of patients/service users that are cared for by your IC services? *[I.e. the general age profile, prevalence of illness, degree of physical disability/independence etc.]*

**Implementation History**

- What do you think initiated the decision for your organisation to use IC as a new model of care? How did it come about? What and/or who were the drivers and facilitators? Are you aware of any initial barriers to the idea and/ or implementation?
- If I were a patient/service user being supported by the IC care program, what differences do you think I would see compared to ‘usual’ care (or care that was provided before the IC services were developed)?

**Context Influence**

- Please list any contextual factors you feel should be considered as facilitators or barriers to IC? What makes them a facilitator or barrier? *[I.e. Political and regulatory context, Financial context, Historical context, Geographical context, Demographics, Workforce, Organisational context (provider market/access and availability; Cultural context: leadership/initiators/ drives/ability to challenge established practice, openness and trust, shared vision and values; normative cultures; political and organisational narratives and expectations etc).*

**Care Intervention and Care Co-ordination**

- What is your understanding of the different components within your organisations IC services?
- Is there a process map or care pathway approach that describes the IC service components within your organisation? *[Please provide us with a document and/or describe this in your own words].*
- Do you have specific documentation regarding how decisions are made within the IC services? [If so, please provide us with it and/or describe this in your own words].
- How are patients/ service users referred into the IC program? *[I.e. are there any specific eligibility criteria? If so, please elaborate].*
- What risk stratification, diagnostic tools, treatment plans, guidance’s and technical resources are used in the referral process? Are these shared across different organisation sites? *[Please elaborate on your answer].*
- Following assessment, how is the care plan undertaken? Who develops the care plan and decides on the appropriate referral*? [I.e. which professionals are involved][*
- Is there shared decision-making on care options with the service user and their carer/family?
- What forms of care do patient/service users receive? *[I.e. Which organisations/professionals provide the different elements of care? Is any of this care provided indirectly (e.g. via a third party)?]*
- Which healthcare professionals are involved in delivering care and at what stage of the patients IC care pathway? *[Please specify].*
- Who ultimately, do you think is in control of making decisions about the treatment and care of the patient/service user? And who takes the overall accountability for co-ordinating this care? *[e.g. named individuals or care co-ordinators; is the accountability shared across the team; are their ‘hand-offs’ of responsibility between partners in care?]*
- Are you aware of any strategies that have been set up to support organisational development and delivery of IC?
- To what extent do the care professionals operate as a team? *[I.e. are there MDTs? If so, who is in the team? How does this operate? How often do the MDTs meet? How do the different professionals communicate with each other? What do they discuss/what decisions are taken?*
- How are you kept informed about developments regarding your IC services? What are the methods or mechanism of knowledge sharing within the MDTs across the different organisation and sites (including social and voluntary services) [*i.e. do you feel this is appropriate – e.g. in terms of the amount and clarity of information and method of communication?]*
- How would you describe the relationship between managers and professionals in the program? In addition, what do you think a good relationship looks like.

- What would you say were the current strengths and weaknesses of the MDT that has been developed between/within the different organisational partners and sites involved in the delivery and support of IC services?
- To what extent is personal continuity of care (i.e. a named care co-ordinator) used in supporting care to the patient/service user? *[I.e. how important do you think is building strong personal relationships with the service user and/or their carers/ family?]*
- What approaches are used to involve patients/service users/carers/ the family in supporting them to self-care? *[I.e. does the program use any specific tools for this - e.g. education, telehealth, peer support etc.]*

**Information systems**

- Looking forward, do you think there are any key issues and challenges for developing and improving information and ICT needs within the delivery of IC? If so, in what ways might these be improved?
- Is your IC work currently supported by the use of ICT?
- In your opinion what are the advantages and drawbacks of using ICT in your work routine?
- Please describe the ICT arrangements within/ between organisational partners involved in the delivery of your IC services?
- What would you like to have that it is not yet available, in terms of ICT support for your IC services?

**Financial Flows**

- What is your understanding of the current finance arrangements to support the organisation, management and delivery of your IC services?
- Do you think that the current finance arrangements could be improved, if so how *[Please specify]*
- In your opinion can IC contribute to a more cost-effective healthcare? *[*Please *give reasons for your answers].*

**Outcomes & Measurements**

- How is the delivery of IC monitored and measured? By who?? *[I.e. What data is collected to measure outcomes/improvements and over what time period?].*
- How do you/the MDTs act on these results and take these findings into account?

**Barriers and Facilitators to Effective Implementation**

- What do you think have been the key challenges/barriers for your organisation to deliver its successful IC services?
- What do you think have been the key facilitators in enabling your organisations IC services to progress and become successful?*[i.e. for both organisational issues and service delivery point of views]*
- Looking ahead, what will be needed to ensure the continued success of your IC services?
- What key lessons have YOU learned to-date in terms of the ability to successfully organise and manage you involvement in the IC services?

**Sustainability and Transferability**

- What are your perceptions of the sustainability of IC? Do you think there could be any challenges in sustainability?
- How easy do you think replication of these types of IC services would be for other types of disease/conditions, and for other organisations?
- Looking forward, what do you think are the key issues and challenges of working across different organisations and sites, in the delivery of IC services? Are there any ways in which this could be improved?

**Concluding remarks**

Thank you for your time and contributions - you will be informed about the overall project results/findings. Before concluding our interview I would like to ask if there anything else you would like to add that you have not mentioned previously.

**End of interview**
